# A DREM-Based Approach for the Identification of Chaotic Systems

**DOI:** 10.3390/e27090971

**Published:** 2025-09-18

**Authors:** Carlos Aguilar-Ibanez, Miguel S. Suarez-Castanon, Belem Saldivar, José E. Valdez-Rodríguez, Eloísa García-Canseco

**Affiliations:** 1 Centro de Investigacion en Computacion, Instituto Politecnico Nacional, Ciudad de Mexico 07738, Mexico; jvaldezr2018@cic.ipn.mx; 2 Escuela Superior de Computo, Instituto Politecnico Nacional, Ciudad de Mexico 07738, Mexico; mssuarez@ipn.mx; 3 Departamento de Control Automatico, Centro de Investigacion y de Estudios Avanzados del Instituto Politecnico Nacional, Ciudad de Mexico 07360, Mexico; belem.saldivar@cinvestav.mx; 4 Facultad de Ciencias, Universidad Autonoma de Baja California, Baja California 22860, Mexico; eloisa.garcia@uabc.edu.mx

**Keywords:** chaotic systems, parameter identification, least-squares method, high-gain observer

## Abstract

A straightforward methodology for identifying certain classes of chaotic systems based on a novel version of the least-squares method, assuming they are algebraically observable and identifiable with respect to a measurable output, is introduced. This output allows us to express the original system as a chain of integrators, where the last term, which depends on the output and its corresponding time derivatives, lumps the system’s non-linearities. We can factorize this term into a regressor function multiplied by an unknown-parameter vector, suggesting that a high-gain observer can be used to simultaneously and approximately estimate the states of the pure integrator and the evolution of the lumped nonlinear term. This allows us to rewrite the original system as a linear regression equation. This configuration enables the above-mentioned least-squares method to recover the chaotic-system parameters.

## 1. Introduction

Chaotic-system identification is a challenging problem that has been studied by physicists, mathematicians, and engineers for at least the last three or four decades. Finding solutions to this problem is necessary because they are needed to solve other actual problems belonging to different disciplines [1,2,3]. For instance, they help us to understand complex phenomena, such as turbulence, fluid dynamics, and weather patterns; to analyze heart rhythms [4], brain activity, and other biological processes that exhibit chaotic behavior; to model and predict unpredictable behaviors in financial markets and economic systems; and to cipher and decipher information in communication engineering [5], to mention just a few areas of application. From a control theory perspective, chaos is important due to its difficulty in predicting complex behavior and its high sensitivity to initial conditions and minor parameter variations. In this context, we can consider the identification of chaotic systems based on measurable output as a fundamental problem because, in several experiments and actual applications, it is impractical, albeit not impossible, to measure the whole state of this kind of system [6]. From a control theory perspective, chaotic-system identification has been tackled through three approaches. The first consists of system inversion [7], where we see the unknown parameters as external inputs, and we consider the available measurable signal to be the system’s output. We aim to find an asymptotic inverse of this mapping (see [7,8] for details). The second approach is based on adaptive control and identification theory (see [9,10,11,12,13,14]). Finally, the third approach is inspired by algebraic methods following the ideas of H. Sira-Ramirez, and M. Fliess introduced in [15,16,17].

Robust adaptive estimation is used in [18] to propose a synchronization method for additive disturbed partially linear systems driven by unknown inputs. The authors of this work, which is based on control theory arguments, provide necessary and sufficient conditions to ensure the estimation of system parameters and information recovery.

To accomplish Duffing mechanical system synchronization and parameter estimation, a Lyapunov-based method is introduced in [19]. To this end, the authors designed a secondary system that asymptotically follows a chosen primary system. Even when secondary–primary synchronization is ensured, temporal parameter convergence is not guaranteed.

A rapid and precise identification algebraic method for Chua’s chaotic oscillator’s unknown constant parameters is developed in [20]. This algebraic method provides an exact formula for the unknown parameters’ accurate calculation, assuming the availability of two measurable output voltage signals.

In [21], the authors developed a chaotic-system parameter estimation dynamic novel strategy. This strategy can use different control functions like Lie algebra, backstepping, or sliding mode. Due to the parameter estimation and feedback interaction, the approach exhibits an adaptive structure.

Based on particle swarm optimization, an parameter identification method for chaotic systems is introduced in [22]. This method has the advantage of being simple to implement and fast in terms of convergence, avoiding extra computational complexity. In [23], the authors combined particle swarm optimization and the dynamic inertia method to identify online Lorenz chaotic-system parameters, using the tools in [24] to propose a similar solution.

Combining a suitable version of the least-squares method and variation calculus, the authors of [25] derived a dynamic system that governs the evolution of all parameters in a chaotic system.

In [26], the authors introduced a new likelihood function that offers a simple and practical approach to parameter estimation in chaotic dynamical systems. To this end, they propose a geometric function that compares the observed data and the output of a parameterized model.

The authors of [27] approached chaotic-system parameter estimation as multi-dimensional optimization by combining a hybrid differential evolution algorithm and an artificial bee colony algorithm.

In [28], the authors combined the Jaya algorithm with the Powell method to propose a new hybrid algorithm, called Jaya–Powell, for Lorenz chaotic-system parameter estimation.

Recently, some new advances in system observations have been introduced. These proposals include Immersion-and-Invariance-based reduced-order observers [29,30,31] and parameter estimation-based observation [32,33,34]. These relatively new approaches allow us to design observers for systems with a highly complex structure. These new approaches can be used to synchronize or observe chaotic systems.

The above brief list of works is not exhaustive, yet we consider these works to be the most relevant and closely related to the solution to the chaotic-system identification problem we introduce in this study.

Here, we propose a straightforward methodology to address the parameter identification problem for chaotic systems that are algebraically observable and identifiable with respect to a single measurable output. This type of system makes it possible to address several scientific problems in a direct manner and with minimal information, such as the design of state observers, the solutions to synchronization problems among multiple systems, and system identification. In addition, identifying chaotic systems is essential because it allows the estimation of parameters that may not be directly accessible or may vary over time in experimental physics, ensures accurate prediction of real system behavior through precise parameter characterization, and enables practical applications such as chaos-based information encryption, which requires real-time knowledge of system parameters. To achieve this, we use a novel version of the least-squares method (LSM) in combination with a model-free high-gain observer, which allows us to estimate the unmeasured variables with high accuracy. To apply our methodology, we must assume that the chaotic system is algebraically observable and identifiable with respect to some suitable output and that its trajectories lie within a compact set. We underscore that we based the parameter estimation on the so-called Dynamic Regressor Extension and Mixing (DREM) procedure [35], even when it does not necessarily require that the system exhibits chaotic behavior. Additionally, the DREM procedure can ensure convergence without assuming persistent excitation, and it is able to detect abrupt changes in the parameter values.

We organize the rest of this work as follows. In Section 2, we introduce the main problem of this work and the needed assumptions. In Section 3, we develop the identification methodology and the corresponding model-free linear observer. In Section 4, we apply the method to solve the output identification of some chaotic systems and present the numerical experiments that allow us to assess the effectiveness of the identification scheme. Finally, in Section 5, we introduce our concluding remarks.

**Notation** **1.** *We represent the identity matrix of dimension n as $I_{n}$ . For symmetric matrices A $\in R^{n\times n}$ and B $\in R^{n\times n}$ , A>B (A≥B) means that A−B is positive (semi-definite).*

### Preamble

Here, we introduce the two useful algebraic properties we use to design our chaotic identification scheme (see [16]).

- Consider a smooth nonlinear system, described by the state vector $X={\left\{x_{i}\right\}}_{1}^{n}\in {ℜ}^{n}$ and by the output vector $Y={\left\{y_{i}\right\}}_{1}^{m}\in {ℜ}^{m}$ , of the form
(1) $$\dot{X}=f\left(X,P\right),\;Y=h\left(X\right),$$
  where h(·) is a smooth vector function and $P\in {ℜ}^{l}$ is a constant parameter vector. Let $Y^{\left(j\right)}$ be the $j^{th}$ time derivative of the vector *Y*. We say that the vector state *X* is **algebraically observable** if it can be uniquely expressed as
(2) $$X=\Phi (Y,\ldots ,Y^{\left(j\right)},P),$$
  for some integer *j* and for some smooth function Φ .
- Moreover, if the vector of parameters, *P*, satisfies the linear relation
(3) $$\Omega_{1}\left(Y,\ldots ,Y^{\left(j\right)}\right)=\Omega_{2}\left(Y,\ldots ,Y^{\left(j\right)}\right)P,$$
  where $\Omega_{1}\left(\cdot \right)$ and $\Omega_{2}\left(\cdot \right)$ are, respectively, n×1 and n×l smooth matrices, then *P* is said to be **algebraically linearly identifiable** with respect to the output vector *Y*.

## 2. Problem Formulation

Consider the following chaotic configuration:

(4) $$\sum :\begin{array}{c} \dot{x}=F\left(t,x,\Theta \right)\\ y=h(x) \end{array}$$

where $x=\left[x_{1},\ldots ,x_{n}\right]\in {ℜ}^{n}$ is the system state, $\Theta \in {ℜ}^{k}$ is a constant parameter vector, y∈ℜ is the single measurable output, and *F* and *h* are functions that depend on the argument $x_{i}$ . We assume that the solution x is forward complete [31,36]. That is, the trajectories starting at time $t_{0}$ are defined for all times $t>t_{0}$ . Let us assume that system (4) can be transformed through iterative derivatives of Lie as follows:

(5) $$\begin{array}{c} {\dot{y}}_{1}=y_{2}\\ :\\ {\dot{y}}_{n}=\phi \left(t,y,\Theta \right). \end{array}$$

where $y_{1}=y$ , and $y\in {ℜ}^{n}$ is defined as

$$y=(y_{1}=h\left(x\right),y_{2}=\dot{h}\left(x\right),\ldots ,y_{n}=h^{n-1}\left(x\right)),$$

and suppose that ϕ can be factorized as

(6) $$\phi =\Lambda^{T}\left(t,y\right)\Theta ,$$

where $\Lambda \in {ℜ}^{k}$ is a computable vector, and $\Theta \in {ℜ}^{k}$ .

Notice that under conditions (5) and (6), we can say that system (4) is algebraically observable and identifiable with respect to the output *y* [16,17,37]. These facts will be referred to as Assumption 1. Please note that, even when this assumption is restrictive, there exist several chaotic systems that satisfy it. Notable examples include Duffing’s system, Lorenz’s systems, Rössler’s system, Chen’s systems, Genesio’s system, and the Van der Pol oscillator [1,37,38]. From now on, we consider a case where the vector y is well-defined and bounded.

**Problem** **1.**
*Let us consider system (4) under Assumption 1. Then, the objective consists of ensuring that the identification error satisfies the following:*

(7) $$\left|\hat{\Theta }-\Theta \right|<\epsilon ,$$

*where $\hat{\Theta }$ is the estimation of *Θ*, and ϵ can be as small as desired, with its size relying on the accuracy estimation of the unknown variables. ${\left\{{\dot{y}}_{i}\right\}}_{i=1}^{n}$ .*

We end this section by introducing the assumption needed to solve the above-mentioned problem.

**Assumption** **1.**
*For every given set of finite initial conditions, a smooth and bounded solution $y\left(t\right)=y_{1}\left(t\right)$ exists for the nonlinear differential Equation (5). Furthermore, the smooth, absolute, and bounded values of the function ϕ(t) are unknown. That is,*

$$\underset{t\geq 0}{\sup}\left|\dot{\phi }\left(t,y\right)\right|\leq \bar{\phi }<\infty .$$

Regarding Assumption 1, we mention that several chaotic systems can be brought to configuration (5), even when the dependency of the parameters is not entirely linear ([1,38]), for example, the systems of Chua, Lorenz, and Chen. We can obtain that configuration using over-parameterization. That is, the vector of parameters can have a dimension greater than *k*; that is, we can use MΘ instead of Θ , where rank(M)=k . On the other hand, based on the generic condition in Assumption 1, we assumed that the chaotic-system trajectories are confined inside a compact set, and we neglected the stationary-state solutions.

**Main advantages of the proposed control methodology**

- Regarding the parametric convergence, even when it is not possible to mathematically prove it, there exists strong evidence that allows us to claim that we can almost always accomplish convergence to the actual parameter values. The latter also holds for non-chaotic behavior.
- The parameter estimation convergence time can be as small as needed if we choose a suitable identification parameter, as we show in the following sections.
- This parameter identification problem has been previously tackled using the gradient descent and the least-squares methods. However, these methods only ensure parameter estimation convergence as time approaches infinity, provided the system exhibits chaotic behavior.

## 3. The Identification Procedure

Here, we introduce the above-mentioned chaotic-system identification procedure inspired by the DREM procedure introduced in [35,39], which, as already mentioned, can ensure convergence without using the strong persistent excitation condition. To this end, we take Equation (6) as

(8) $$\phi =\Lambda^{T}\left(t,y\right)\Theta ,$$

where $\phi ={\dot{y}}_{n}\in R$ and the regressor function $(\Lambda^{T}t,y)$ $\in R^{k}$ are known and bounded functions of time, and $\Theta \in R^{k}$ is a constant unknown vector. Then, we first introduce k−1 linear stable operators, $H_{i}:L_{\infty }\to L_{\infty }$ , with i={1,2,…,k−1} , where we fix the output, for any bounded input, as

(9) $${\left(\right)}_{f_{i}}=\left[H_{i}\left(\cdot \right)\right]\left(t\right),$$

where the filter $H_{i}$ has the following form:

(10) $$H_{i}\left(p\right)=\frac{\alpha_{i}}{p+\kappa_{i}},$$

with $p=\frac{d}{dt}$ and $\alpha_{i}\neq 0$ , $\kappa_{i}>0$ . Applying the above-defined operator (10) to the previously defined regressor (8), we obtain the new filtered regression:

$${\left\{\phi \right\}}_{f_{i}}={\left\{\Lambda^{T}\left(t,y\right)\right\}}_{f_{i}}\Theta +\mu_{i},$$

where $\mu_{i}$ exponentially converges to zero (in the following development, we set $\mu_{i}=0$ , as suggested in [35,40]). Once again, we apply this operation k−1 times to construct the following system of equations:

(11) $$Y_{e}\left(t\right)=M_{e}\left(t\right)\Theta ,$$

where

(12) $$\begin{array}{ccc} Y_{e}\left(t\right)=\left[\begin{array}{c} \phi \\ {\left\{\phi \right\}}_{f_{1}}\\ :\\ {\left\{\phi \right\}}_{f_{k-1}} \end{array}\right] & ; & M_{e}=\left[\begin{array}{c} \Lambda^{T}\left(t,y\right)\\ {\left\{\Lambda^{T}\left(t,y\right)\right\}}_{f_{1}}\\ :\\ {\left\{\Lambda^{T}\left(t,y\right)\right\}}_{f_{k-1}} \end{array}\right]. \end{array}$$

Now, we first multiply (11) by the adjunct matrix $M_{e}$ , and we obtain *k* scalar regressors of the form

$$Y_{i}\left(t\right)=\delta \left(t\right)\theta_{i},$$

with i={1,2,…,k} , where

(13) $$\begin{array}{ccc} \delta \left(t\right)=\det\{M_{e}\left(t\right)\} & ; & Y\left(t\right)=adj\left\{M_{e}\left(t\right)\right\}Y_{e}\left(t\right), \end{array}$$

is defined. From the above and using the traditional least-squares method, we can propose the estimation of variable Θ as follows:

(14) $$\dot{\hat{\Theta }}=\delta \left(t\right)\Gamma \left(Y-\delta \left(t\right)\hat{\Theta }\right),$$

where $\Gamma =\Gamma^{T}>0$ . Notice that system (14) can be rewritten in terms of the identification error; that is, we obtain the following equivalent system:

(15) $$\dot{\tilde{\Theta }}=-\delta^{2}\left(t\right)\Gamma \tilde{\Theta },$$

where $\tilde{\Theta }=\hat{\Theta }-\Theta$ . To ensure the convergence of the proposed estimator (14), we use the following Lyapunov function:

$$V=\frac{1}{2}{\tilde{\Theta }}^{T}\Gamma^{-1}\tilde{\Theta },$$

where

$$\dot{V}=-\delta^{2}\left(t\right){∥\tilde{\Theta }∥}^{2},$$

which can be upper-bounded by

$$\dot{V}\leq -{\bar{\lambda }}_{\left\{\Gamma^{-1}\right\}}\delta^{2}\left(t\right)V.$$

From the above, we determine that

$$V\leq V\left(0\right)\exp\left(-{\bar{\lambda }}_{\left\{\Gamma^{-1}\right\}}\int_{0}^{t}\delta^{2}\left(s\right)ds\right),$$

which leads us to

(16) $${∥\tilde{\Theta }∥}^{2}\leq \sqrt{\frac{V(0)}{{\underline{\lambda }}_{\left\{\Gamma^{-1}\right\}}}}\exp\left(-\frac{{\bar{\lambda }}_{\left\{\Gamma^{-1}\right\}}}{2}\int_{0}^{t}\delta^{2}\left(s\right)ds\right),$$

where

$${\underline{\lambda }}_{\left\{\Gamma^{-1}\right\}}I_{k}\leq \Gamma^{-1}\leq {\bar{\lambda }}_{\left\{\Gamma^{-1}\right\}}I_{k}.$$

From inequality (16), we can ensure that $\tilde{\Theta }\to 0$ , as long as t→∞ , if $\delta \left(t\right)\notin L_{2}$ .

We summarize the above discussion in the following proposition:

**Proposition** **1.**
*Consider system (4) under Assumption 1, and the linear regressor (8), where ϕ and Λ(t,y) are known bounded functions of time, and* Θ *is a vector of unknown parameters. If we use k−1 linear $L_{\infty }$ stable operators $H_{i}:L_{\infty }\to L_{\infty }$ i={1,…,k−1} to check if (10) holds, define matrix $M_{e}$ and vector $Y_{e}$ as given in (12), and consider the estimator (14) with δ(t) and Y(t) defined in (13), then the identification error is $\tilde{\Theta }\to 0$ as long as t→∞ , provided $\delta \left(t\right)\notin L_{2}$ .*

**Remark** **1.**
*Notice that the proposed methodology does not have singularities. That is, the equation*

$$adj\left\{A\right\}A=\det\left(A\right)I_{k}$$

*is well-defined even if A is not full-rank. On the other hand, the condition $\delta \left(t\right)\notin L_{2}$ is weaker than the persistency of excitation (see [35,39] for details). Also, we do not need to compute the inverse of a matrix, as is usually needed in algebraic methods.*

**Model-free observer for the estimation of unknown variables**

Because the time derivatives of output *y* are non-available, we introduce the model-free observer based on the linear high-gain Luenberger observer (see [1,41,42]); this kind of observer does not require knowledge of the chaotic dynamic equation; this condition is always fulfilled because we only consider a case where vectors x , y $\in D\subset R^{n}$ , where *D* is a compact set. In other words, the solutions to system (4) are forward complete and uniformly bounded. Boundedness is a common assumption in the observer design literature, yet it holds for many physical systems, such as oscillators or chaotic systems [18].

Let us introduce the following proposition:

**Proposition** **2.**
*Under Assumption 1, for a system of the form (5), the high-gain observer is as follows:*

(17) $$\begin{array}{c} {\dot{\hat{y}}}_{1}={\hat{y}}_{2}-w_{0}\beta_{1}\left({\hat{y}}_{1}-y_{1}\right)\\ {\dot{\hat{y}}}_{2}={\hat{y}}_{3}-w_{0}^{2}\beta_{2}\left({\hat{y}}_{1}-y_{1}\right)\\ :\\ {\dot{\hat{y}}}_{n+1}=-w_{0}^{n+1}\beta_{n+1}\left({\hat{y}}_{1}-y_{1}\right) \end{array}$$

*where $\beta_{i}$ , i={1,2,…,n+1}=I are selected such that the roots of polynomial*

$$p\left(s\right)=s^{n}+\beta_{1}s^{n-1}+\cdots +\beta_{n}s+\beta_{n+1}$$

*are in the open left-half complex plane and $w_{0}>>1$ . Then, the system (17) asymptotically exponentially reconstructs, via the observer’s vector variables ${\hat{y}}_{e}={\left[{\hat{y}}_{1},{\hat{y}}_{2},\ldots ,{\hat{y}}_{n+1}\right]}^{T}$ to the actual vector variables $y_{e}={\left[y_{1},y_{2},\ldots ,y_{n},\phi \right]}^{T}$ , while the observation error ${\tilde{y}}_{e}=y_{e}-{\hat{y}}_{e}$ satisfies*

(18) $$\left|y_{k}-{\hat{y}}_{k}\right|\leq \frac{∥P∥\bar{\phi }}{2w_{0}^{n+2-k}},$$

*for k∈I , and P>0 is a solution to the Lyapunov equation:*

(19) $$P\underline{B}+{\underline{B}}^{T}P=I_{n+1},$$

*with $\underline{B}\in R^{\left(n+1)\times (n+1\right)}$ defined as*

(20) $$\underline{B}=\left[\begin{array}{cccccc} -\beta_{1} & 1 & 0 & \ldots & \; & 0\\ -\beta_{2} & 0 & 1 & \ldots & \; & 0\\ : & \; & & \; & & \\ : & \; & & \; & & \\ -\beta_{n+1} & \; & & \; & & 0 \end{array}\right]$$

**Proof.**  From Equations (5) and (17), it is easy to see that dynamic error observation satisfies the following equation:

(21) $${\dot{\tilde{y}}}_{e}=B{\tilde{y}}_{e}+E_{n+1}\dot{\phi }\left(t,y\right)$$

where $B\in R^{\left(n+1)\times (n+1\right)}$ and $E_{n+1}\in R^{1\times (n+1)}$ are defined as

$$\begin{array}{ccc} B=\left[\begin{array}{cccccc} -\beta_{1}w_{0} & 1 & 0 & \ldots & \; & 0\\ -\beta_{2}w_{0}^{2} & 0 & 1 & \ldots & \; & 0\\ : & \; & & \; & & \\ : & \; & & \; & & \\ -\beta_{n+1}w_{0}^{n} & \; & & \; & & 0 \end{array}\right] & ; & E_{n+1}=\left[\begin{array}{c} 0\\ 0\\ :\\ :\\ 1 \end{array}\right] \end{array}$$

From system Equation (21), we can easily conclude that the observation error is bounded, since *B* is Hurtwitz, and $\dot{\phi }$ is bounded by the assumption. However, to obtain a good estimation error, we introduce the following coordinate change:

(22) $${\tilde{x}}_{e}=diag\left\{w_{0},w_{0}^{2},\ldots ,w_{0}^{n},w_{0}^{n+1}\right\}{\tilde{y}}_{e}$$

which transforms system (21) in the following equation:

(23) $${\dot{\tilde{x}}}_{e}=w_{0}\underline{B}\tilde{x}+w_{0}^{-(n+1)}E_{n+1}\dot{\phi }\left(*\right),$$

where *B* is defined in (20). Now, we propose the needed Lyapunov equation:

$$V_{0}=\frac{1}{2}{\tilde{x}}_{e}^{T}P{\tilde{x}}_{e}$$

where P>0 satisfies the Equation (19). Therefore, the time derivative of $V_{0}$ around the system Equation (23) leads us to

$${\dot{V}}_{0}=-w_{0}{∥{\tilde{x}}_{e}∥}^{2}+w_{0}^{n+1}{\tilde{x}}_{e}^{T}PE_{n+1}\dot{\phi }\left(*\right),$$

which can be upper-bounded by

$${\dot{V}}_{0}\leq -w_{0}{∥{\tilde{x}}_{e}∥}^{2}+w_{0}^{-(n+1)}∥{\tilde{x}}_{e}∥∥P∥\bar{\phi }$$

From the last equation, we can conclude that there exists $t_{0}>0$ , such that

$$∥{\tilde{x}}_{e}∥\leq \frac{∥P∥\bar{\phi }}{w_{0}^{n+2}}$$

Hence, incorporating the definition of (1) into the last inequality, we have

$$\frac{1}{w^{k}}\left|y_{k}-{\hat{y}}_{k}\right|\leq ∥{\tilde{x}}_{e}∥\leq \frac{∥P∥\bar{\phi }}{w_{0}^{n+2}}$$

for k∈I , which coincides with the bound error given in (18). □

**Remark** **2.**
*Because the accuracy of estimates of output-related phase variables depends on the proposed observer gains, it is well-known that these estimates exhibit initial peakings. We can easily overcome this drawback by using a smooth clutching operation on the needed variables, as suggested in [1]. Also, if the system’s flat output y is perturbed by additive noise with mean 0, we can overcome this drawback by filtering this output using, for instance, a high-pass filter. Afterwards, we can apply the time derivative observer approach. We illustrate this procedure in the following section describing the numerical simulations.*

## 4. Output Identification of Some Oscillatory Systems

To test the effectiveness of our control scheme, we designed three numerical simulations to accomplish the parameter identification of an equal number of well-known chaotic systems: the Duffing oscillator, the Genesio system, and the Van der Pol oscillator.

### 4.1. Duffing System

Consider the traditional Duffing mechanical oscillator (**DMO**) described by

$$\begin{array}{c} {\dot{y}}_{1}=y_{2}\\ {\dot{y}}_{2}=-p_{2}y_{2}-p_{3}y_{1}^{3}-p_{1}y_{1}+p_{4}\cos(wt) \end{array}$$

where $y_{1}$ and $y_{2}$ are the oscillator position and velocity, respectively; $p_{3}$ is the magnitude forcing function; *w* is the forcing frequency; γ is the damping coefficient; and $p_{1}$ and $p_{2}$ are the stiffness constants related to the nonlinear spring. It is well-known that the above system exhibits periodic and chaotic behavior when its parameters are in the neighborhood of $p_{1}=-0.4$ , $p_{2}=1.1$ , $p_{3}=2.5$ , w=0.8 , and $p_{4}=1.5$ (see [3]). Under the assumption that the position variable $y_{1}$ is measurable and the frequency *w* is known, we aimed to recover the parameters { $p_{1},p_{2},p_{3},p_{4}$ }. Evidently, due to the DMO configuration, we can claim that it is algebraically observable with respect to the measurable output *y*. That is, it satisfies (5) and (6).

To achieve the above, we propose an observer for the estimation of the following unknown Duffing’s system variables:

(24) $$\begin{array}{c} {\dot{\hat{y}}}_{1}={\hat{y}}_{2}-w_{0}\beta_{1}\left({\hat{y}}_{1}-y_{1}\right)\\ {\dot{\hat{y}}}_{2}={\hat{y}}_{3}-w_{0}^{2}\beta_{2}\left({\hat{y}}_{1}-y_{1}\right)\\ {\dot{\hat{y}}}_{3}=-w_{0}^{3}\beta_{3}\left({\hat{y}}_{1}-y_{1}\right) \end{array}$$

with $\beta_{i}$ , i={1,2,3} selected, such that $p\left(s\right)=s^{3}+\beta_{1}s^{2}+\beta_{2}s+\beta_{3}$ is Hurtwitz. Evidently, ${\hat{y}}_{e}={\left[{\hat{y}}_{1},{\hat{y}}_{2},{\hat{y}}_{3}\right]}^{T}$ is the estimation of $y_{e}={\left[y_{1},y_{2},\phi \right]}^{T}$ , with

$$\phi =-p_{2}y_{2}-p_{3}y_{1}^{3}-p_{1}y_{1}+p_{4}\cos\left(wt\right).$$

Recall that the parameter *w* is given. Then, we adapt the proposed identification procedure. To this end, we must note that

$$\begin{array}{ccc} {\hat{y}}_{3}=\hat{\phi } & \Lambda \left(t,\hat{y}\right)=\left[\begin{array}{c} -y_{1}\\ -{\hat{y}}_{2}\\ -y_{1}^{3}\\ cos(wt) \end{array}\right] & \Theta =\left[\begin{array}{c} p_{1}\\ p_{2}\\ p_{3}\\ p_{4} \end{array}\right] \end{array}$$

with the corresponding filter matrices given by

$$\begin{array}{ccc} Y_{e}\left(t\right)=\left[\begin{array}{c} {\hat{y}}_{3}\\ {\left\{{\hat{y}}_{3}\right\}}_{f_{1}}\\ {\left\{{\hat{y}}_{3}\right\}}_{f_{2}}\\ {\left\{{\hat{y}}_{3}\right\}}_{f_{3}} \end{array}\right] & ; & M_{e}=\left[\begin{array}{c} \Lambda^{T}\left(t,\hat{y}\right)\\ {\left\{\Lambda^{T}\left(t,\hat{y}\right)\right\}}_{f_{1}}\\ {\left\{\Lambda^{T}\left(t,\hat{y}\right)\right\}}_{f_{1}}\\ {\left\{\Lambda^{T}\left(t,\hat{y}\right)\right\}}_{f_{3}} \end{array}\right] \end{array}$$

We take $\delta =\det\{M_{e}\}$ and $Y=adj\left\{M_{e}\right\}Y_{e}$ and substitute this data into the evolution equation for parameter identification $\dot{\hat{\Theta }}=\delta \Gamma \left(Y-\delta \hat{\Theta }\right)$ , where matrix Γ and the filter parameters are given by

(25) $$\begin{array}{c} \Gamma =diag\{40,40,40,40\}\\ \begin{array}{ccc} \alpha_{1}=0.5 & \alpha_{2}=1 & \alpha_{3}=1.2 \end{array}\\ \begin{array}{ccc} \kappa_{1}=5 & \kappa_{2}=2 & \kappa_{3}=1 \end{array} \end{array}$$

**Figure 1:**
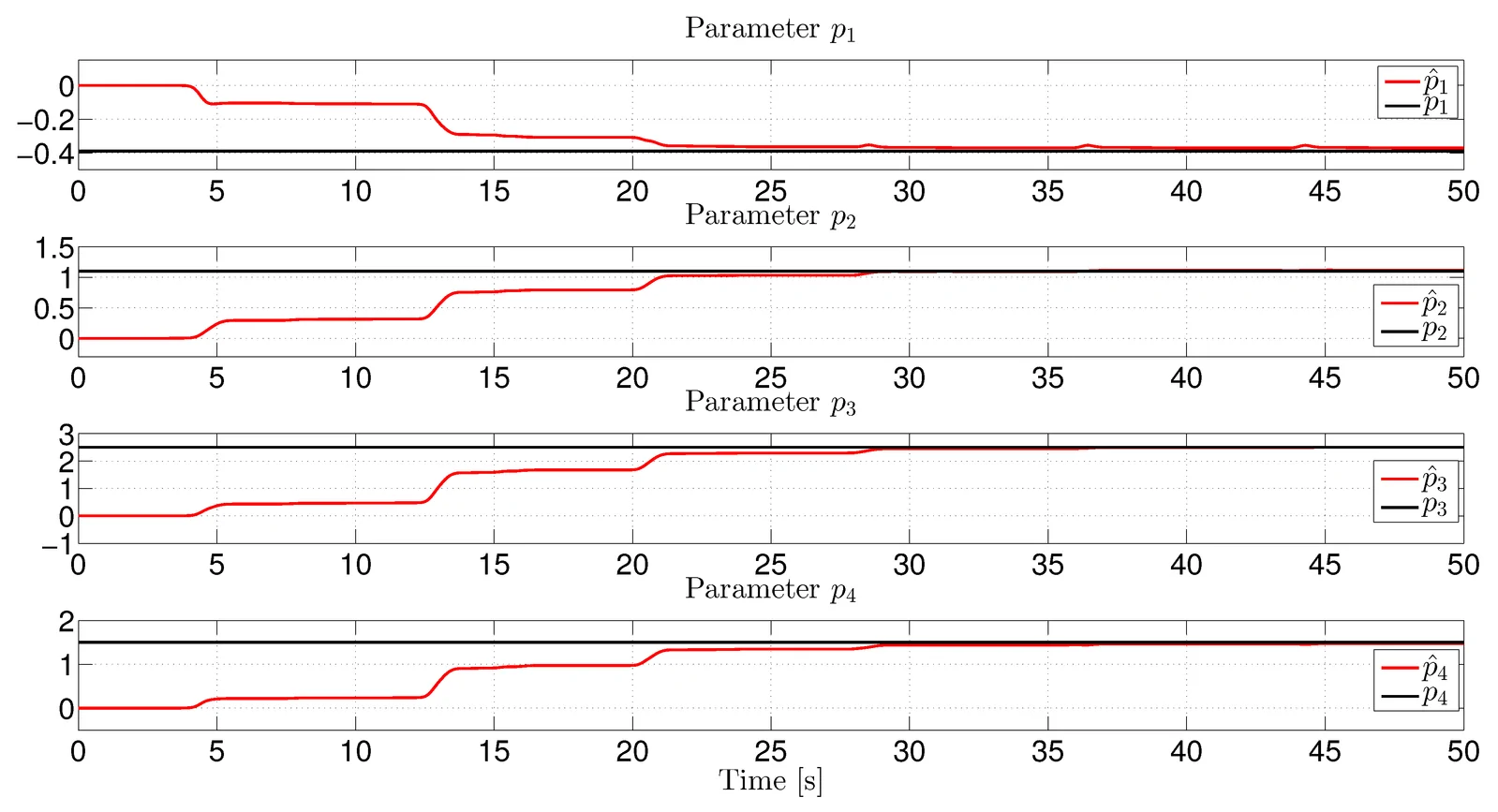
Estimation of unknown DMO parameters.

**Figure 2:**
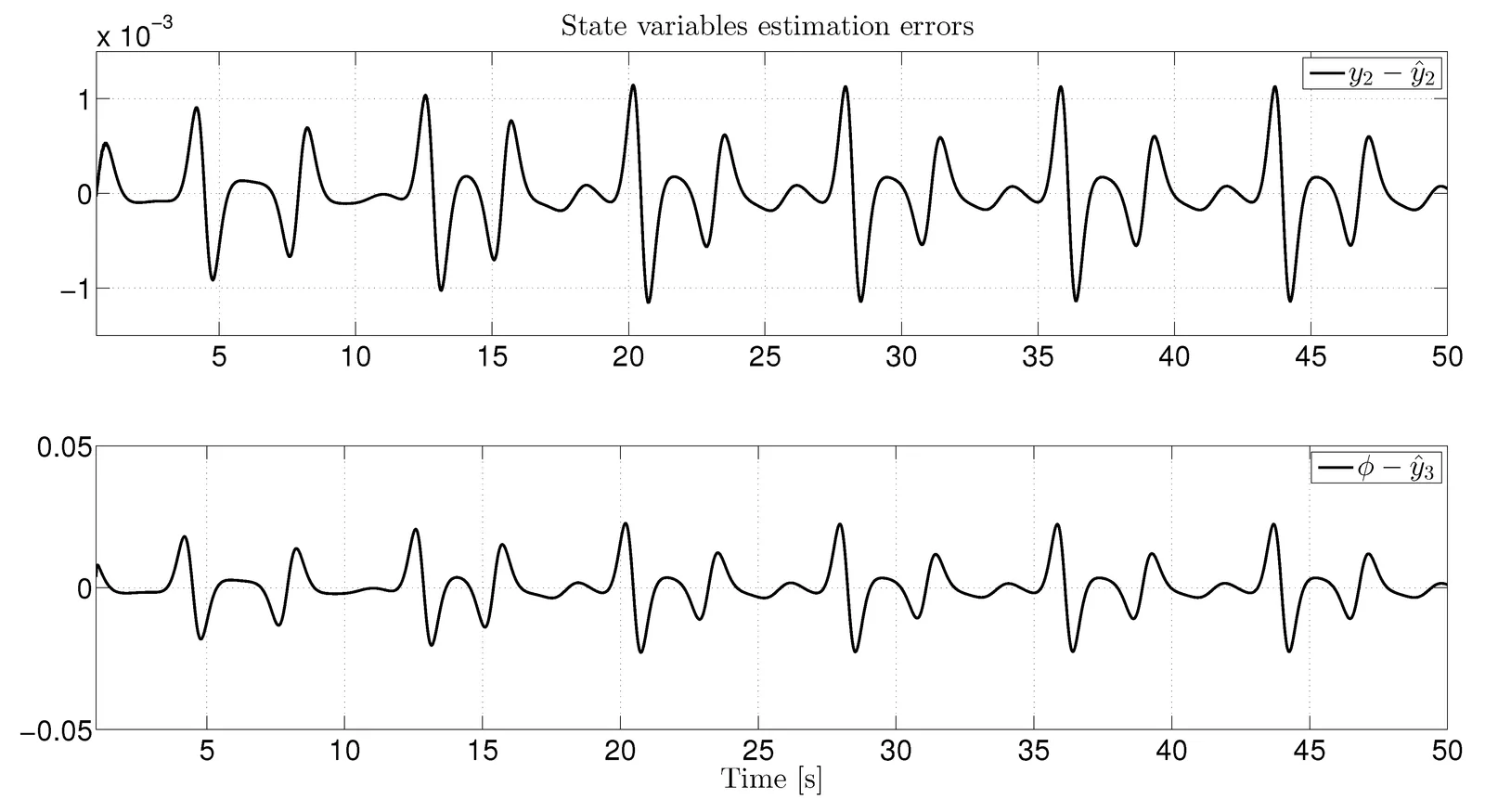
Estimation of DMO state variable errors.

Now, to carry out the identification of the unknown parameters of the DMO, we set the initial conditions of the observer system and the identification system at the origin, while we set that of the DMO at $y_{1}\left(0\right)=1$ and $y_{2}\left(0\right)=-1$ . We fixed the DMO parameters at $p_{1}=-0.4$ , $p_{2}=1.1$ , $p_{3}=2.5$ , $p_{4}=1.5$ , and w=0.8 , and we chose the observer gain parameters so that the associated characteristic polynomial was given by $p\left(s\right)={\left(s+1\right)}^{3}$ with $w_{0}=70$ . We show the corresponding simulation results in Figure 1 and Figure 2. In Figure 1, we can see that the identification process exhibits good performance. That is, after 30 s, the estimated parameters converge very close to the following values:

$$\begin{array}{cccc} {\hat{p}}_{1}\approx -0.4001 & {\hat{p}}_{2}\approx 1.105 & {\hat{p}}_{3}\approx 2.49 & {\hat{p}}_{4}=1.49 \end{array}$$

In Figure 2, we can see the observation errors of the following variables:

$$\begin{array}{ccc} {\tilde{y}}_{2}=y_{2}-{\hat{y}}_{2} & ; & {\tilde{y}}_{3}=\phi -{\hat{y}}_{3}. \end{array}$$

In this figure, we notice that, after 1 s, both estimation errors converge to a rather small vicinity around the origin in the order of $10^{-3}$ and $10^{-2}$ for ${\tilde{y}}_{2}$ and ${\tilde{y}}_{3}$ , respectively. From these simulations, we can conclude that the proposed identification schema allows us to recover all the parameters reasonably well, even when the actual variables $y_{2}$ and ϕ are not measurable.

### 4.2. Genecio System

The Genesio system may be considered the simplest third-order chaotic system, and its dynamics are given by the following set of differential equations [43]:

$$\begin{array}{c} {\dot{y}}_{1}=y_{2}\\ {\dot{y}}_{2}=y_{3}\\ {\dot{y}}_{2}=-p_{1}y_{1}-p_{2}y_{2}-p_{3}y_{3}+p_{4}y_{1}^{2} \end{array}$$

where $y_{1}$ , $y_{2}$ , and $y_{3}$ are the state variables, with measurable output $y=y_{1}$ , and $p_{1}$ , $p_{2}$ , $p_{3}$ , and $p_{4}$ are unknown positive quantities satisfying $p_{2}p_{3}<p_{3}$ . For this experiment, we consider that the fixed parameters $p_{1}$ , $p_{2}$ , $p_{3}$ , and $p_{4}$ present an abrupt change defined as follows:

$$\begin{array}{ccc} p_{1}=\left\{\begin{array}{c} 5\;\;\;\mathrm{for}\;\;t\leq \;30\\ 6\;\;\;\mathrm{for}\;\;t>30 \end{array}\right. & \; & p_{2}=\left\{\begin{array}{c} 2.92\;\;\;\mathrm{for}\;\;t\leq \;30\\ 2.5\;\;\;\mathrm{for}\;\;t>30 \end{array}\right.\\ p_{3}=\left\{\begin{array}{c} 1\;\;\;\mathrm{for}\;\;t\leq \;30\\ 2\;\;\;\mathrm{for}\;\;t>30 \end{array}\right. & \; & p_{4}=\left\{\begin{array}{c} 0.8\;\;\;\mathrm{for}\;\;t\leq \;30\\ 1.2\;\;\;\mathrm{for}\;\;t>30 \end{array}\right. \end{array}$$

To estimate unknown time derivatives of *y*, we use the following observer:

$$\begin{array}{c} {\dot{\hat{y}}}_{1}={\hat{y}}_{2}-w_{0}\beta_{1}\left({\hat{y}}_{1}-y\right)\\ {\dot{\hat{y}}}_{2}={\hat{y}}_{3}-w_{0}^{2}\beta_{2}\left({\hat{y}}_{1}-y\right)\\ {\dot{\hat{y}}}_{3}={\hat{y}}_{4}-w_{0}^{3}\beta_{3}\left({\hat{y}}_{1}-y\right)\\ {\dot{\hat{y}}}_{4}=-w_{0}^{4}\beta_{4}\left({\hat{y}}_{1}-y\right) \end{array}$$

with $\beta_{i}$ selected such that

$$s^{4}+\beta_{1}s^{3}+\beta_{2}s^{2}+\beta_{3}s+\beta_{4}={\left(s+1\right)}^{4}$$

and where ${\hat{y}}_{e}={\left[{\hat{y}}_{1},{\hat{y}}_{2},{\hat{y}}_{3},{\hat{y}}_{4}\right]}^{T}$ is the estimation of $y_{e}={\left[y_{1},y_{2},y_{3},\phi \right]}^{T}$ , with

$$\phi =-p_{1}y_{1}-p_{2}y_{2}-p_{3}y_{3}+p_{4}y_{1}^{2}.$$

Then, we carry out the identification procedure as follows:

$$\begin{array}{ccc} {\hat{y}}_{4}=\hat{\phi } & \Lambda \left(t,\hat{y}\right)=\left[\begin{array}{c} -y_{1}\\ -{\hat{y}}_{2}\\ -{\hat{y}}_{3}\\ y_{1}^{2} \end{array}\right] & \Theta =\left[\begin{array}{c} p_{1}\\ p_{2}\\ p_{3}\\ p_{4} \end{array}\right] \end{array}$$

with the corresponding filter matrices given by

(26) $$\begin{array}{ccc} Y_{e}\left(t\right)=\left[\begin{array}{c} {\hat{y}}_{4}\\ {\left\{{\hat{y}}_{4}\right\}}_{f_{1}}\\ {\left\{{\hat{y}}_{4}\right\}}_{f_{2}}\\ {\left\{{\hat{y}}_{4}\right\}}_{f_{3}} \end{array}\right] & ; & M_{e}=\left[\begin{array}{c} \Lambda^{T}\left(t,\hat{y}\right)\\ {\left\{\Lambda^{T}\left(t,\hat{y}\right)\right\}}_{f_{1}}\\ {\left\{\Lambda^{T}\left(t,\hat{y}\right)\right\}}_{f_{1}}\\ {\left\{\Lambda^{T}\left(t,\hat{y}\right)\right\}}_{f_{3}} \end{array}\right]. \end{array}$$

**Figure 3:**
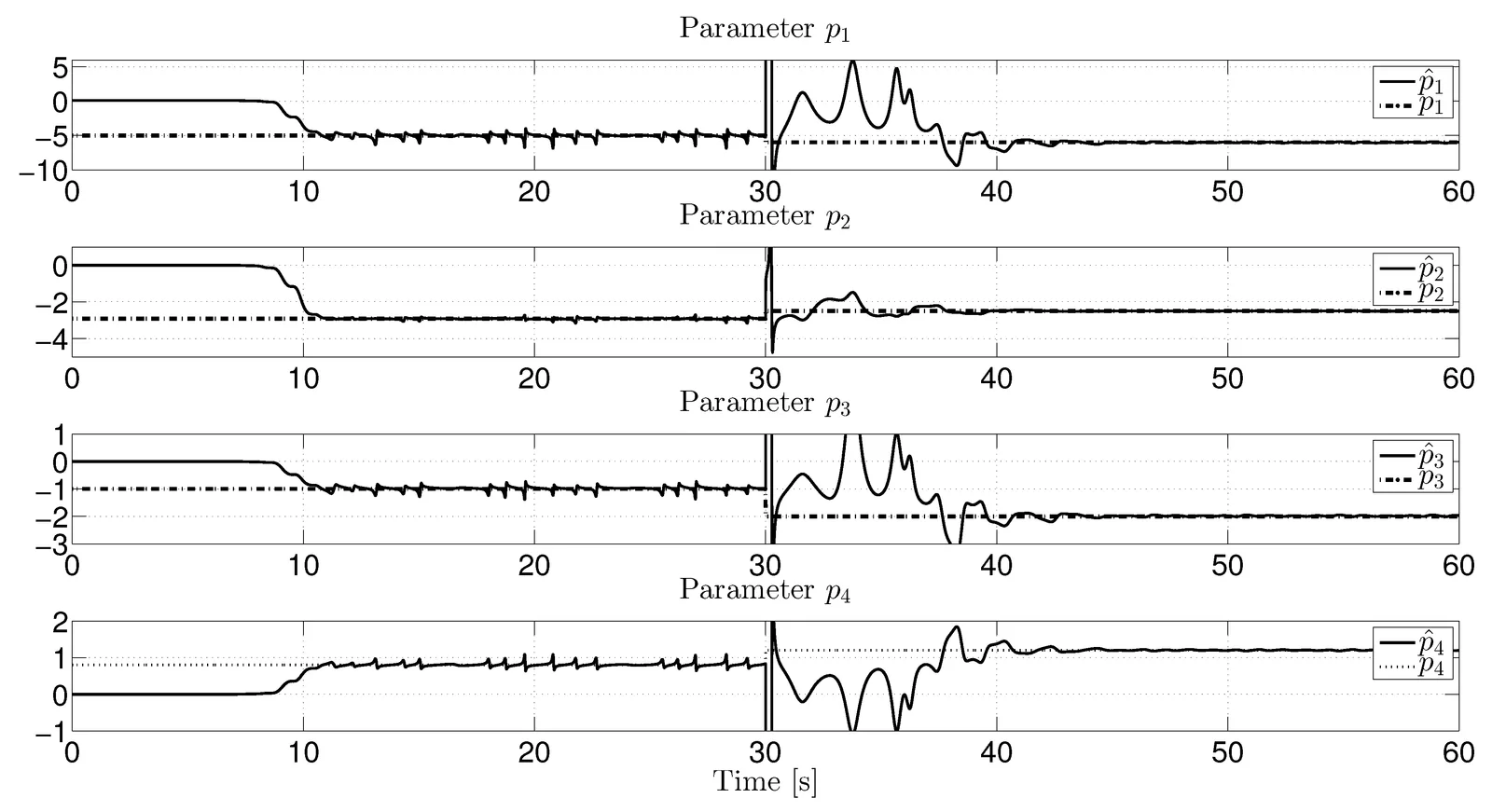
Parameter estimation for the Genesio system when the parameters are abruptly changed after 30 s.

**Figure 4:**
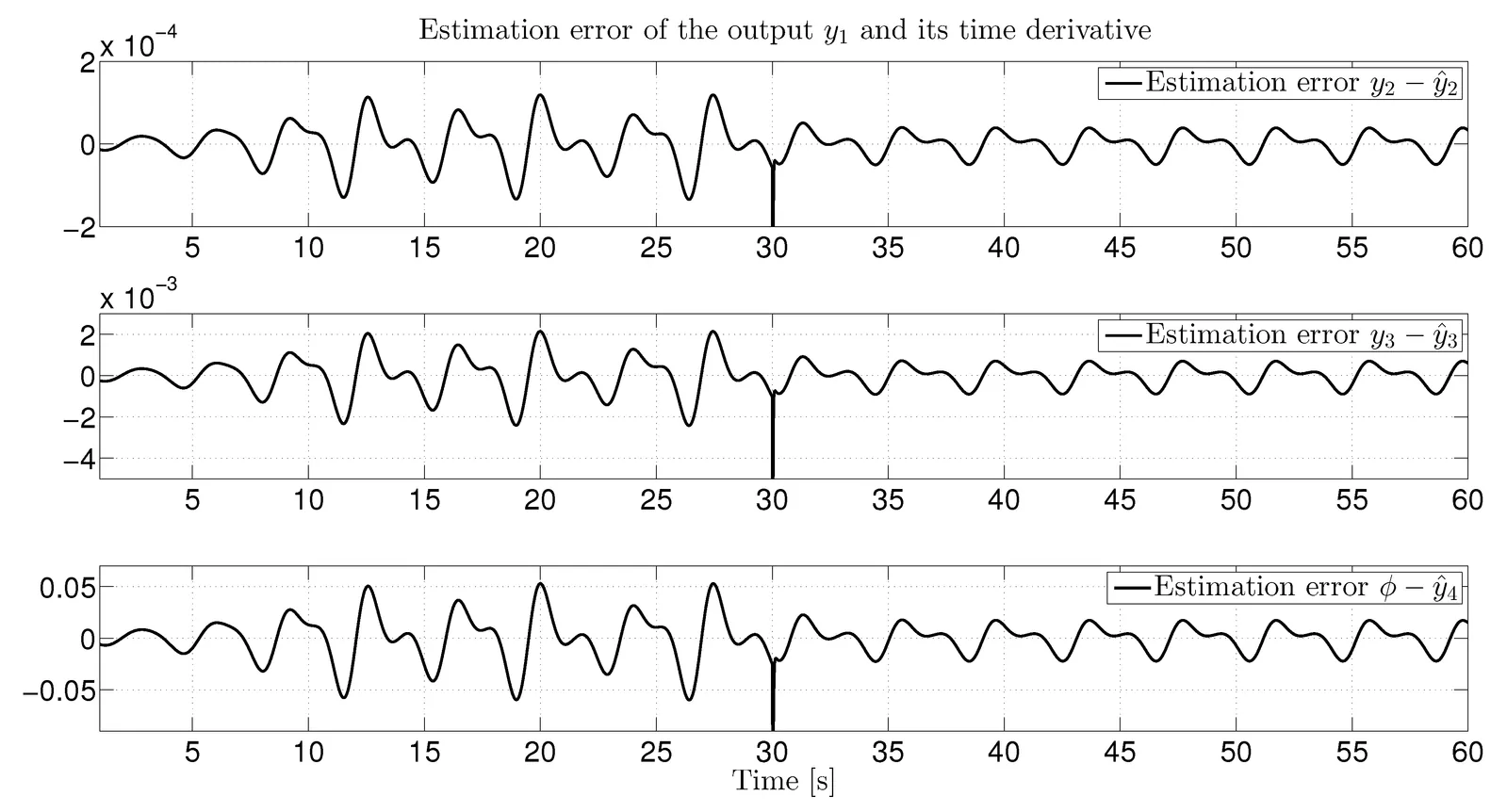
State estimation error for the Genesio system when the values of its parameters are abruptly changed after 30 s.

Finally, using the filter matrices (26) in (13) and (14), we obtained the evolution of the parameter set $\hat{\Theta }$ , which we show in Figure 3. From this figure, it is evident that even in the presence of small abrupt changes in the parameter values, our estimator is capable of detecting these changes with an estimation error in the order of $10^{-2}$ . In Figure 4, we show the observation errors of the following variables:

$$\begin{array}{ccc} {\tilde{y}}_{2}=y_{2}-{\hat{y}}_{2} & {\tilde{y}}_{3}=y_{3}-{\hat{y}}_{3} & {\tilde{y}}_{4}=\phi -{\hat{y}}_{4} \end{array}$$

We show the evolution of these errors in Figure 4, where we can see that they are in the orders of $10^{-4}$ , $10^{-3}$ , and $10^{-2}$ for ${\tilde{y}}_{2}$ , ${\tilde{y}}_{3}$ , and ${\tilde{y}}_{4}$ , respectively. Once again, we can see that all the observation errors converge to a small vicinity around the origin.

### 4.3. The Van der Pol oscillator

Consider the Van der Pol oscillator described by the following set of equations:

$$\begin{array}{c} {\dot{y}}_{1}=y_{2}\\ {\dot{y}}_{2}=-p_{1}y_{2}-p_{2}y_{1}^{2}y_{2}-p_{3}y_{1} \end{array}$$

where $y_{1}$ and $y_{2}$ are the oscillator’s position and velocity, respectively; $p_{1}$ and $p_{2}$ are parameters that control the damping strength; and $p_{3}$ is a parameter related to the elastic constant. In the case when $p_{3}=p_{2}=-p_{1}=1$ , which has been widely studied, the system presents a limit cycle. In this numerical experiment, we assume that the measurable variable is perturbed by additive noise with zero mean and a uniform distribution, denoted by $y_{m}\left(t\right)=y\left(t\right)+n\left(t\right)$ , where n∈[−0.2,0.2] . To improve the performance of the proposed method, we use the following simple filter:

$$\begin{array}{c} {\dot{z}}_{1}=z_{2}\\ {\dot{z}}_{2}=-2\zeta w_{e}z_{2}-w_{e}^{2}\left(z_{1}-y_{m}\right) \end{array}$$

where $\zeta =1/\sqrt{2}$ is the damping coefficient, and $w_{e}$ is the natural frequency of the filter. For simplicity, we set $w_{e}=10$ . Once the filtered signal $z_{1}\left(t\right)$ is obtained, it is substituted into the observer proposed in (24). That is, we simply replace $y_{1}\left(t\right)$ with $z_{1}\left(t\right)$ . Once again, ${\hat{y}}_{e}={\left[{\hat{y}}_{1},{\hat{y}}_{2},{\hat{y}}_{3}\right]}^{T}$ is the estimation of $y_{e}={\left[y_{1},y_{2},\phi \right]}^{T}$ , with

$$\phi =-p_{1}{\hat{y}}_{2}-p_{2}{\hat{y}}_{1}^{2}{\hat{y}}_{2}-p_{3}{\hat{y}}_{1}.$$

Then, we adapt the proposed identification procedure. To this end, we must note that

$$\begin{array}{ccc} {\hat{y}}_{3}=\hat{\phi } & \Lambda \left(t,\hat{y}\right)=\left[\begin{array}{c} -{\hat{y}}_{2}\\ -{\hat{y}}_{1}^{2}{\hat{y}}_{2}\\ -y_{2} \end{array}\right] & \Theta =\left[\begin{array}{c} p_{1}\\ p_{2}\\ p_{3} \end{array}\right] \end{array}$$

with the corresponding filter matrices given by

$$\begin{array}{ccc} Y_{e}\left(t\right)=\left[\begin{array}{c} {\hat{y}}_{3}\\ {\left\{{\hat{y}}_{3}\right\}}_{f_{1}}\\ {\left\{{\hat{y}}_{3}\right\}}_{f_{2}} \end{array}\right] & ; & M_{e}=\left[\begin{array}{c} \Lambda^{T}\left(t,\hat{y}\right)\\ {\left\{\Lambda^{T}\left(t,\hat{y}\right)\right\}}_{f_{1}}\\ {\left\{\Lambda^{T}\left(t,\hat{y}\right)\right\}}_{f_{1}} \end{array}\right] \end{array}$$

We define $\delta =\det\{M_{e}\}$ and $Y=adj\left\{M_{e}\right\}Y_{e}$ and substitute these definitions into the equation

$$\dot{\hat{\Theta }}=\delta \Gamma \left(Y-\delta \hat{\Theta }\right)$$

where matrix Γ=diag{40,40,40} and the filter parameters are given by the set of { $\alpha_{1}=1,\alpha_{2}=2,\kappa_{1}=2,\kappa_{2}=5$ }.

**Figure 5:**
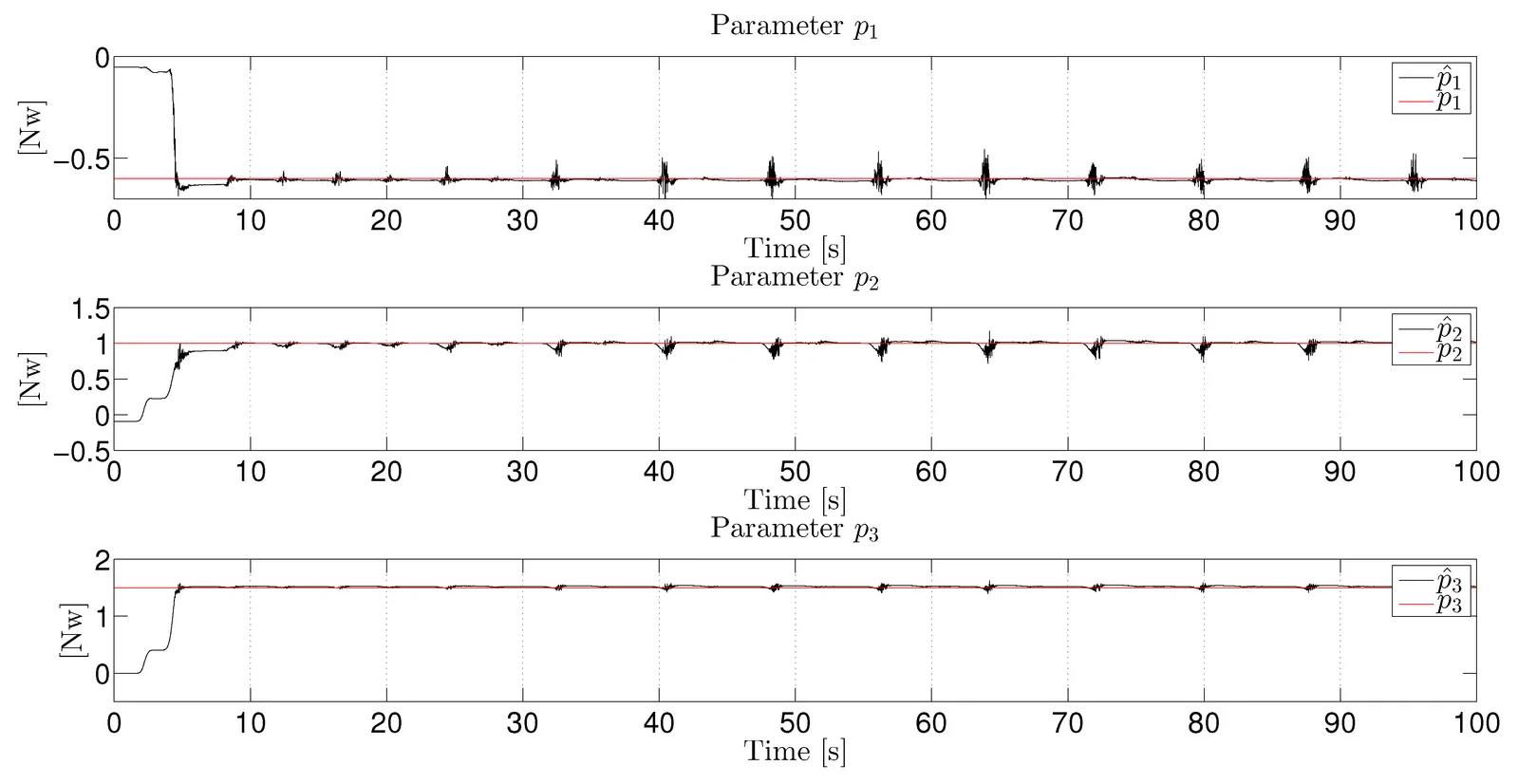
Parameter estimation for the Van der Pol oscillator when its measurable output is perturbed by additive noise.

**Figure 6:**
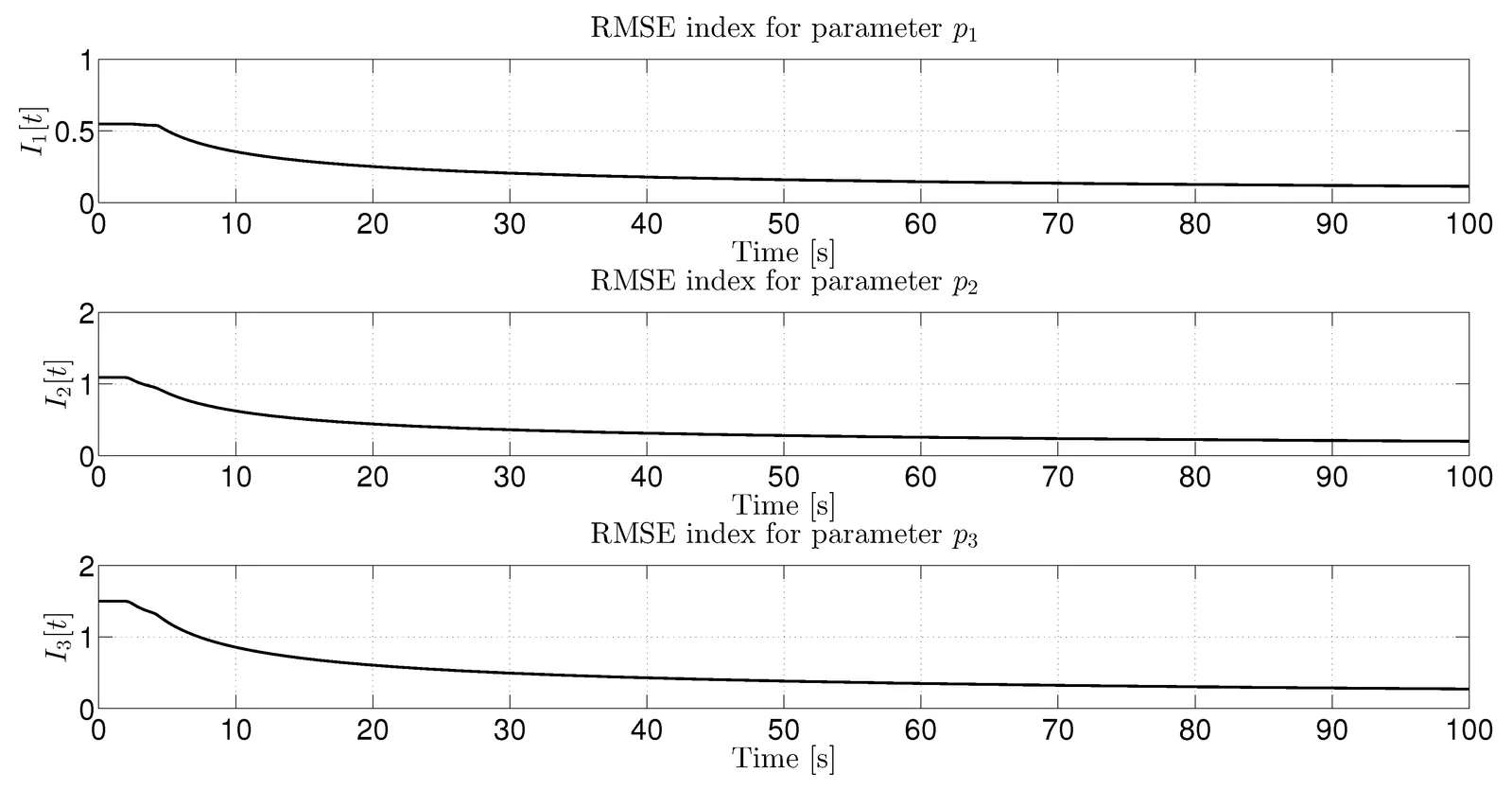
Van der Pol oscillator parameter RMSE estimation when its measurable output is perturbed by additive noise.

To identify the unknown parameters of the Van der Pol system, we set the initial conditions of the observer and identification systems to the origin, and the oscillator system parameters to $y_{1}\left(0\right)=0.26$ and $y_{2}\left(0\right)=-0.5$ . We set the Van der Pol system parameters to $p_{1}=-0.6$ , $p_{2}=1$ , $p_{3}=1.5$ ; we choose the same observer gain parameters as in the first experiment. The simulation results are shown in Figure 5 and Figure 6. In Figure 5, we can see that the identification process shows good performance even when the measurable output is altered by additive noise. After 30 s, the estimated parameters converge very close to the following values:

$$\begin{array}{ccc} {\hat{p}}_{1}\approx -0.58 & {\hat{p}}_{2}\approx 1.105 & {\hat{p}}_{3}\approx 1.49 \end{array}$$

In Figure 6, we can see the corresponding Root-Mean-Square Error metrics, which were computed using the following formula:

$$I_{i}\left[t\right]=\sqrt{\frac{1}{T}\int_{0}^{T}{\left({\hat{p}}_{i}\left(s\right)-p_{i}\right)}^{2}ds}$$

In this figure, we notice that, after 50 s, the RMSE parameter estimations approach the values $I_{1}\left[t\right]⋍0.11$ , $I_{1}\left[t\right]⋍0.18$ , and $I_{1}\left[t\right]⋍0.17$ . Please note that this oscillator has been identified even when it does not exhibit chaotic behavior.

Finally, in Table 1, we summarize the parameter estimation errors to facilitate a quick comparison and to gauge the performance of our approach.

**Remark** **3.**
*We consider that we can considerably improve the estimations of variables ϕ and *Γ* if we use the exact same differentiators as the ones proposed by Levant in [44,45], with the inconvenience of needing to know the Lipschitz constant for each chaotic system. On the other hand, as far as we know, there are no rules regarding tuning the filter parameters for the DREM method. In the case of higher-order systems, we can use an extended version of the GPIA-based observer proposed in [1] to increase the accuracy of higher-order time derivative computations; however, this accuracy demands more computing time. Additionally, if the number of parameters increases, the number of cofactors that need to be estimated also increases, along with a corresponding increment in calculation time. If that were not enough, we can face an ill-conditioned adjacent matrix Y in (13). Conversely, with fewer unknown parameters, our approach is more accurate. The interested reader can find a detailed description of the above in [35].*

## 5. Conclusions

In this paper, we presented a novel procedure for identifying chaotic systems, assuming they are algebraically observable and identifiable with respect to a suitable output. This procedure makes it possible to express them as a chain of integrators perturbed by a nonlinear term that lumps the system output nonlinearities. This term is a function of the output and its respective derivatives, which we can factorize as a regressor function multiplied by the vector of unknown parameter of the chaotic system. This configuration suggests that we should introduce a high-gain observer to simultaneously estimate the states of the pure integration system and the evolution of the lumped nonlinear term. This observer allows us to approximately estimate the nonlinear system state variables and the unknown perturbation input. On the other hand, we can express the original system in terms of a linear regression equation, where we can approximately obtain the regression function and the perturbation estimation. Then, we can apply a new version of the least-squares or DREM technique to recover the chaotic system’s unknown-parameter vector accurately within a very short convergence time. We conduct an analysis of convergence of the observer and identifier schemes based on Lyapunov theory arguments. Note that the proposed identification method has the advantage of being easy to numerically implement, only needing to know the single output and the system structure. We illustrated our methodology via three well-known oscillatory systems. The digital computer simulation allows us to claim that our methodology accurately solves the chaotic-system identification problem in a relatively short time.

## Figures and Tables

**Table 1:** Table to compare the parameter estimation errors of the Duffing, Genesio, and Van der Pol oscillators.

Parameter Estimation Errors				
**Oscillatory System**	$p_{1}-\tilde{p_{1}}$	$p_{2}-\tilde{p_{2}}$	$p_{3}-\tilde{p_{3}}$	$p_{4}-\tilde{p_{4}}$
Duffing	−0.0205	−0.01369	0.260	0.3699
Genesio	0.067	−0.08	−0.132	0.013
Van der Pol	0.009	−0.0093	−0.01	N/A

## Data Availability

The original contributions presented in this study are included in the article. Further inquiries can be directed to the corresponding author.
